# NH_3_ Sensor Based on 3D Hierarchical Flower-Shaped *n*-ZnO/*p*-NiO Heterostructures Yields Outstanding Sensing Capabilities at ppb Level

**DOI:** 10.3390/s20174754

**Published:** 2020-08-22

**Authors:** Zhenting Zhao, Haoyue Yang, Zihan Wei, Yan Xue, Yongjiao Sun, Wenlei Zhang, Pengwei Li, Weiping Gong, Serge Zhuiykov, Jie Hu

**Affiliations:** 1 Guangdong Provincial Key Laboratory of Electronic Functional Materials and Devices, Huizhou University, Huizhou 516001, Guangdong, China; zhzhting@hzu.edu.cn (Z.Z.); gwp@hzu.edu.cn (W.G.); 2 Center of Nano Energy and Devices, College of Information and computer, Taiyuan University of Technology, Taiyuan 030024, Shanxi, China; yanghaoyue0220@link.tyut.edu.cn (H.Y.); xueyan0179@link.tyut.edu.cn (Y.X.); sunyongjiao@tyut.edu.cn (Y.S.); zhangwenlei@tyut.edu.cn (W.Z.); lipengwei@tyut.edu.cn (P.L.); 3 Department of Solid State Science, Faculty of Science, Ghent University Global Campus, 119 Songdomunhwa-ro, Yeonsu-gu, Incheon 21985, Korea; zihan.wei@ugent.be (Z.W.); serge.zhuiykov@ghent.ac.kr (S.Z.)

**Keywords:** hierarchical structures, NH**_3_** sensing properties, *n*-ZnO/*p*-NiO heterojunction, gas sensor, ammonia

## Abstract

Hierarchical three-dimensional (3D) flower-like *n*-ZnO/*p*-NiO heterostructures with various Zn_x_Ni_y_ molar ratios (Zn**_5_**Ni**_1_**, Zn**_2_**Ni**_1_**, Zn**_1_**Ni**_1_**, Zn_1_Ni**_2_** and Zn**_1_**Ni**_5_**) were synthesized by a facile hydrothermal method. Their crystal phase, surface morphology, elemental composition and chemical state were comprehensively investigated by XRD, SEM, EDS, TEM and XPS techniques. Gas sensing measurements were conducted on all the as-developed Zn_x_Ni_y_-based sensors toward ammonia (NH**_3_**) detection under various working temperatures from 160 to 340 °C. In particular, the as-prepared Zn**_1_**Ni**_2_** sensor exhibited superior NH**_3_** sensing performance under optimum working temperature (280 °C) including high response (25 toward 100 ppm), fast response/recovery time (16 s/7 s), low detection limit (50 ppb), good selectivity and long-term stability. The enhanced NH**_3_** sensing capabilities of Zn**_1_**Ni**_2_** sensor could be attributed to both the specific hierarchical structure which facilitates the adsorption of NH**_3_** molecules and produces much more contact sites, and the improved gas response characteristics of *p-n* heterojunctions. The obtained results clear demonstrated that the optimum *n*-ZnO/*p*-NiO heterostructure is indeed very promising sensing material toward NH**_3_** detection for different applications.

## 1. Introduction

It is common knowledge that ammonia (NH**_3_**) is one of the most widely produced industrial chemicals, which plays an important role in manufacturing systems such as dyes, drugs, plastics, etc. [1]. Moreover, NH**_3_** is also easily found in our daily life, including the dairy and ice cream plants, wineries and breweries, and juice and drink processing [2]. However, it should be noticed that NH**_3_**, as strongly irritating gas, is not only flammable under the concentrations of 16–28 vol % in air, but also exhibit highly toxic and hazardous effects on the ecosystem and human health [3,4,5]. For humans, the acute exposure to NH**_3_** may cause severe health problems, such as headaches, nausea, and intense burning of the skin, eyes, throat and nose. Moreover, if interactions with high concentration of gaseous NH**_3_** take place for a long time, people will suffer from serious pharyngeal pain, chest tightness, dyspnea, toxic phenomena and even death [6]. According to the recommendation of National Institute for Occupational Safety and Health (NIOSH), the threshold limit value of NH**_3_** should be below 25 ppm in the work environments place [2]. Therefore, it is highly desirable and essential to develop high performance sensors for NH**_3_** detection at the low concentration level for industrial, agricultural and health monitoring applications.

Recently, metallic oxide semiconductor (MOS), such as ZnO [7], SnO**_2_** [8], NiO [9], WO**_3_** [10], In**_2_**O**_3_** [11], etc. have extensively studied for constructing gas sensors, because of their advantages of environmental protection, low price, fast response speed to flammable and organic volatile gases [12,13]. Nevertheless, the most of MOS based sensors still face many drawbacks such as low sensitivity, high working temperature and poor reliability and selectivity, which might block their industrial applications. Thus, to improve further gas sensing performance of the MOS based sensors, different approaches were adopted and implemented including but not limited to the morphology controlling [14,15], doping with suitable nanometals [16,17] and formation heterojunctions and heterointerfaces [18,19]. Among these methods, heterojunction construction, which forms at the interface between the different MOS, is one of the most effective strategies for enhancement of gas sensing properties. Moreover, construction of heterojunction semiconductor provides an important way to combine the different properties of each single component into one combined system [20]. In addition, the heterostructured MOS based sensing materials have also the features of finer grains, enhanced surface-to-volume ratio and increased gas accessibility.

Zinc oxide (ZnO), as a typical *n*-type semiconductor sensing material, has attracted great attention because of its good gas sensing performance [7,21,22]. Up to now, many working groups tried to improve the gas sensing properties of ZnO-based gas sensors by construction *p*-*n* heterojunctions. For example, Chen et al. reported the fabrication of ultraporous ZnO/NiO heterojunction networks using flame synthesis and aerosol self-assemble, and studied their gas sensitivity toward volatile organic compounds (VOC) gas [23]. The reported results demonstrated that the ZnO/NiO nanoparticle networks demonstrated excellent sensitivity to acetone and ethanol gas. Kaur et al. fabricated NiO/ZnO one-dimensional nanowire, in which the NiO nanowires act as backbones for the growth of ZnO [24]. The hybrid nanostructures showed enhanced VOC gas sensing behavior compared to NiO sensors. Moreover, Zhou et al. prepared the nanodisk-shaped NiO/ZnO-based SO**_2_** gas sensor, and the obtained gas response can reach to 16.25 [25]. Notwithstanding many previous works reporting great gas sensing properties of ZnO/NiO heterojunction structures for detection of the different gases, to the best of our knowledge, the optimization Zn:Ni content and its corresponding sensing performance towards NH**_3_** has not yet been done. Therefore, there is much room for the synthesis and development of *n*-ZnO/*p*-NiO based NH**_3_** sensors with superior sensing properties.

In the present work, we report systematic studies with respect to the effect of ZnO/NiO *p*-*n* heterojunctions on the NH**_3_** sensing. For this purpose, a series of *n*-ZnO/*p*-NiO hierarchical structures with different Zn:Ni molar ratios (Zn_x_Ni_y_) were successfully synthesized using the one-pot hydrothermal route and then systematically characterized. Gas sensing measurements were carried out on all the as-developed Zn_x_Ni_y_-based sensors towards NH**_3_** detection. After comprehensive studies, it was found that the different heterojunction proportions can greatly affect the gas-sensing performance. The measured results proved and confirmed that the as-prepared Zn**_1_**Ni**_2_** sensor exhibited the best gas sensing performance for NH**_3_** at the selected optimum working temperature of 280 °C, and consequently, demonstrated that the 3D *n*-ZnO/*p*-NiO heterostructure is a very promising sensing material for NH**_3_** sensors with superior capabilities at low ammonia concentrations.

## 2. Materials and Methods

### 2.1. Chemicals and Reagents

Zinc acetate dihydrate (Zn(CH**_3_**COO)**_2_**·2H**_2_**O, ≥ 99.9%), Nickel(II) acetate tetrahydrate (Ni(CH**_3_**COO)**_2_**·4H**_2_**O, ≥ 99.0%), Glycine (NH**_2_**CH**_2_**COOH, ≥ 99.0%), and Ammonium bicarbonate (NH**_4_**HCO**_3_**, ≥ 99.0%) were purchased from Sigma-Aldrich (Shanghai, China). All chemical reagents in the experiments were of analytical grade and used as received without further purification. The deionized water (DI water, 18.25 MΩ·cm) used in all experiments was prepared with a Milli-Q water purification system (Milli-Q HX 7000, Merck KGaA, Darmstadt, Germany).

### 2.2. Synthesis of ZnO/NiO Hierarchical Structures

3D hierarchical structures of *n*-ZnO/*p*-NiO heterojunctions were synthesized by one-pot hydrothermal method. Typically, 16 mmoL NH**_4_**HCO**_3_** was dissolved into 40 mL DI water for formation an aqueous solution. Then 16 mmol NH**_2_**CH**_2_**COOH, different molar ratio of Zn(CH**_3_**COO)**_2_**·2H**_2_**O and Ni(CH**_3_**COO)**_2_**·4H**_2_**O (Zn:Ni = 5:1, 2:1, 1:1, 1:2, 1:5) were dissolved into another 40 mL DI water with magnetic stirring for 1 h at the room temperature. After that, the obtained NH**_4_**HCO**_3_** solution was added into the above solution, and transferred into 100 mL Teflon-lined stainless-steel autoclave. Then, after 3 h of reaction at 180 °C, the resulting product was collected via centrifugation, washing and drying. Finally, the obtained product was annealed at 500 °C for 2 h in air for structural stabilization. Additionally, the 3D hierarchical flower-like n-ZnO/p-NiO heterostructures with the different Zn:Ni molar ratios were also synthesized for comparison and marked as Zn**_5_**Ni**_1_**, Zn**_2_**Ni**_1_**, Zn**_1_**Ni**_1_**, Zn**_1_**Ni**_2_** and Zn**_1_**Ni**_5_**, respectively. The as-synthesized samples were stored in the dry environment at room temperature for subsequent experiments.

### 2.3. Material Characterization 

The structure and the phase of the sample were evaluated by X-ray diffraction (XRD, D/MAX-2500 V/PC, Rigaku, Japan). The microstructures of as-synthesized 3D flower-like *n*-ZnO/*p*-NiO hierarchical structures were analyzed by scanning electron microscopy (SEM, JSM-7100F, JEOL, Japan) and their crystalline structures were further confirmed by transmission electron microscopy (TEM, JEM-2100, JEOL, Japan). Furthermore, the surface compositions and chemical state of samples were examined by X-ray photoelectron spectroscopy (XPS, ESCALAB 250Xi, Thermo Fisher Scientific, Waltham, MA, USA).

### 2.4. Gas Sensors Fabrication and Measurement

**Figure 1:**
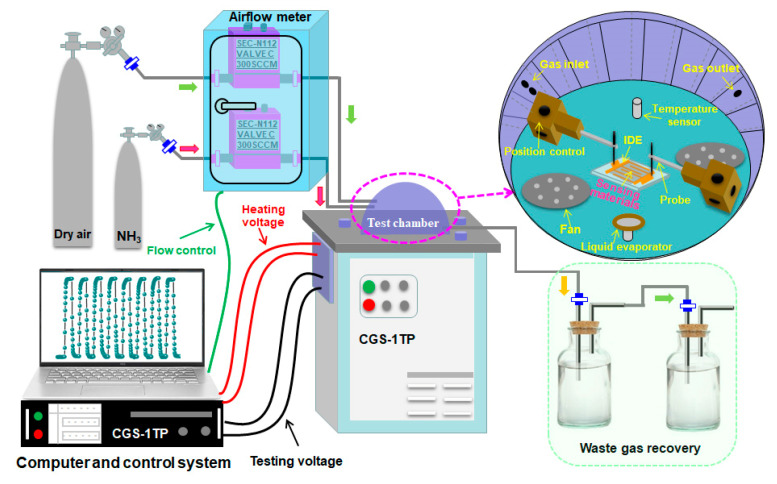
Schematic diagram of gas sensors testing system.

The gas sensors were fabricated by pasting the as-synthesized 3D flower-like *n*-ZnO/*p*-NiO hierarchical structures on the interdigitated electrode (IDE). The IDE was prepared by making interdigital gold electrode on the glass substrate by photolithography and magnetron sputtering techniques. To improve its stability, the *n*-ZnO/*p*-NiO modified IDE-based sensing electrodes were transferred to aging stage with 5 V for 3 days. Chemiresistive properties of the 3D *n*-ZnO/*p*-NiO heterostructures were evaluated using CGS-1TP intelligent analysis system (Elite, Beijing, China) under the laboratory conditions (30 ± 3% RH, 25 ± 2 °C). The schematic diagram of the gas sensor testing system used in our investigation is shown in Figure 1. The response is defined as Ra/Rg (Ra is the sensor resistance in air and Rg reflects the resistance in the targeted gas). We defined the time of the sensor to reach 90% of the total resistance change as the response and recovery time (τ_res_ and τ_rec_), respectively.

## 3. Results

### 3.1. Structure and Morphology Characterization

**Figure 2:**
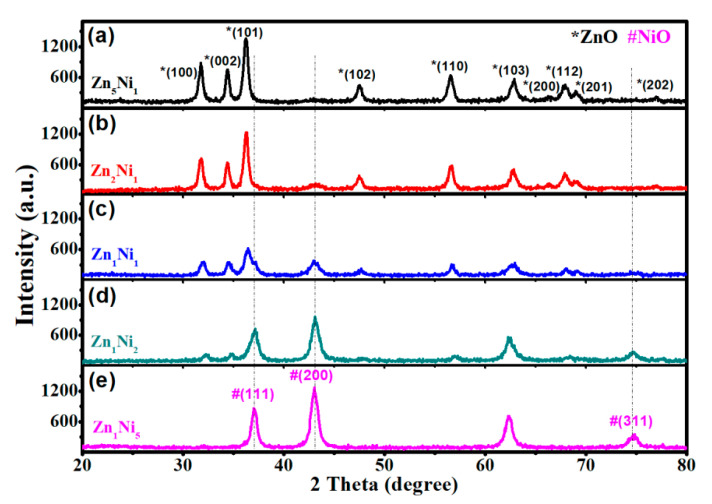
XRD patterns of the 3D *n*-ZnO/*p*-NiO hierarchical structures with the different molar ratios of Zn and Ni: Zn**_5_**Ni**_1_** (**a**), Zn**_2_**Ni**_1_** (**b**), Zn**_1_**Ni**_1_** (**c**), Zn**_1_**Ni**_2_** (**d**), and Zn**_1_**Ni**_5_** (**e**).

The crystal structures of as-synthesized 3D flower-like *n*-ZnO/*p*-NiO nanocomposites were investigated by XRD and the main results are depicted in Figure 2a, where all the diffraction peaks marked with (*) can be indexed to the patterns of ZnO (JCPDS-36-1451). And the peaks located at 2θ = 31.68°, 34.42°, 36.25°, 37.45°, 56.57°, 62.81°, 66.30°, 67.92°, 69.05° and 76.95° can be indexed to the (100), (002), (101), (102), (110), (103), (200), (112), (201) and (202) planes of ZnO. However, with the increasing Ni molar ratio Figure 2b–e, the XRD patterns of NiO (marked with (#)) appeared for Zn**_2_**Ni**_1_**, Zn**_1_**Ni**_1_**, Zn**_1_**Ni**_2_** and Zn_1_Ni_5_ samples. The planes can be ascribed to the standard XRD patterns of NiO (JCPDS-47-1049). Meanwhile, the intensity of diffraction peaks related to (111), (200) and (311) planes of NiO get stronger and sharper with the increasing proportion of Ni, confirming the existence of NiO in the heterostructures. Moreover, there are no other characteristic peaks were detected for all *n*-ZnO/*p*-NiO hierarchical structures, and thus the obtained results demonstrated high purity of as-synthesized samples.

**Figure 3:**
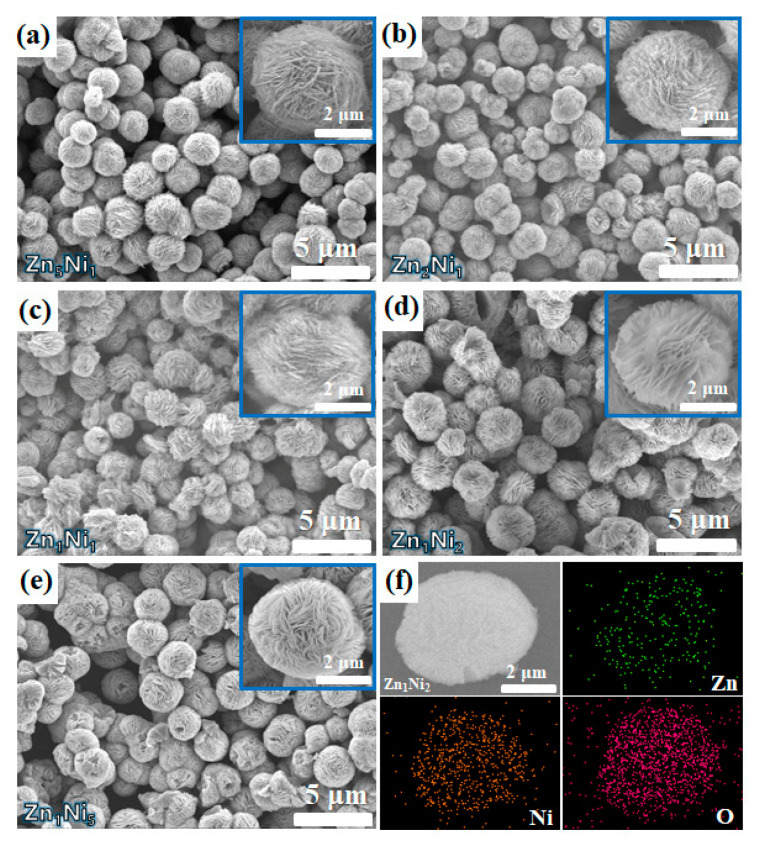
SEM images of as-prepared samples: Zn**_5_**Ni**_1_** (**a**), Zn**_2_**Ni**_1_** (**b**), Zn**_1_**Ni**_1_** (**c**), Zn**_1_**Ni**_2_** (**d**) and Zn**_1_**Ni**_5_** (**e**). (**f**) The elemental mapping of Zn**_1_**Ni**_2_** sample.

The morphology of all the as-synthesized 3D *n*-ZnO/*p*-NiO hierarchical structure was investigated by scanning electron microscopy (SEM). Figure 3a–e shows the SEM images of the *n*-ZnO/*p*-NiO composites, which were prepared by the different Zn-to-Ni molar ratio. It appears to indicate that all samples possess 3D flower-like hierarchical microstructure. The diameter of the individual flower is about 3.5 ± 0.5 μm. Furthermore, the insets in Figure 3a–e show the high magnification SEM images of the single nanostructure. It can be observed that the flower-like *n*-ZnO/*p*-NiO is self-assembled by aggregation of many nanosheets, and the Zn**_1_**Ni**_2_** possesses the largest distance between the nanosheets, which will facilitate exposure to the target gas. Moreover, the elemental composition of Zn**_1_**Ni**_2_** hierarchical structures was determined using EDS analysis. Specifically, Figure 3f shows the elemental mapping images of a typical Zn**_1_**Ni**_2_** hierarchical structure, clearly representing the distribution of Zn, Ni, and O elementals in the 3D flower-like structure.

**Figure 4:**
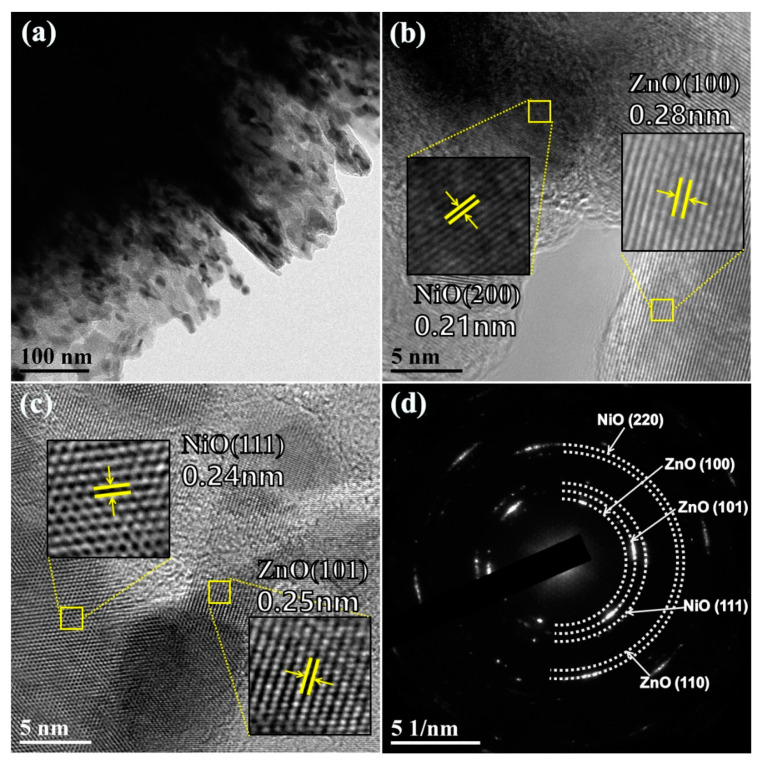
(**a**) Low resolution TEM image of Zn**_1_**Ni**_2_** hierarchical structures. (**b**,**c**) High resolution TEM images of Zn**_1_**Ni**_2_** hierarchical structures. (**d**) Selected area electron diffraction (SAED) patterns of Zn**_1_**Ni**_2_** hierarchical structures.

To clearly observe the morphology structure of as-synthesized n-ZnO/p-NiO hierarchical structure, transmission electron microscopy (TEM) analysis was conducted on the Zn**_1_**Ni**_2_** sample. Figure 4a shows the morphological features at the boundary of nanosheets structure, which displays the uneven edges. The microstructure of Zn**_1_**Ni**_2_** hierarchical structure was further studied by the high resolution-TEM (HRTEM). The measured lattice fringes presented in Figure 4b clearly exhibited two different spacings of approximately 0.21 and 0.28 nm, which can be assigned to the (200) plane of NiO and (100) plane of ZnO, respectively. Similarly, the interplanar distances of 0.24 and 0.25 nm agree well with the lattice spacing of the (111) plane of cubic NiO and (101) plane of hexagonal ZnO Figure 4c. Moreover, the selective area electron diffraction (SAED) pattern Figure 4d revealed that the diffraction rings can be indexed as the (100), (101), (110) planes of hexagonal ZnO and the (111), (220) planes of cubic NiO. Thus, the obtained TEM results were very consistent with previous XRD analysis and elemental mapping images, demonstrating that the 3D *n*-ZnO/*p*-NiO hierarchical structures have been successfully synthesized using hydrothermal method.

**Figure 5:**
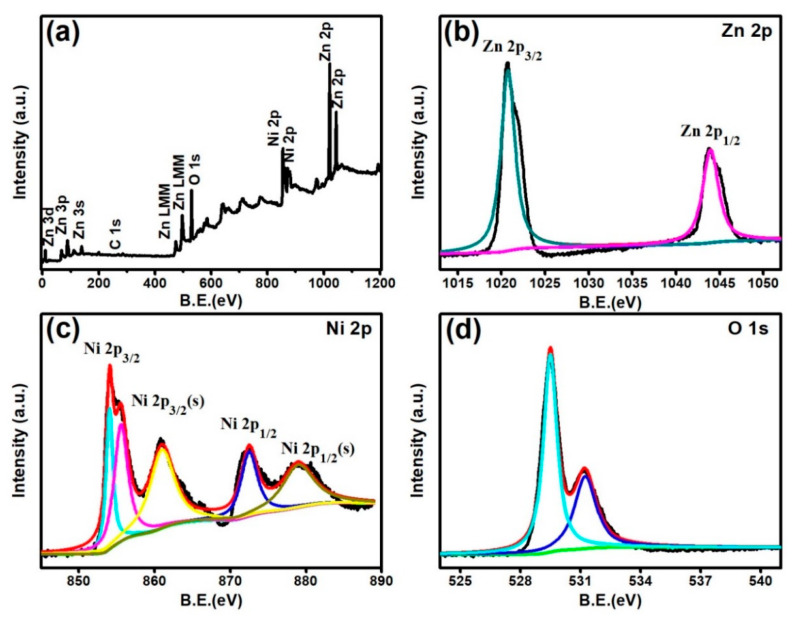
XPS survey spectra of Zn**_1_**Ni**_2_** (**a**), high resolution spectra of Zn 2p (**b**), Ni 2p (**c**) and O 1s (**d**).

Furthermore, to determine the elemental composition and chemical binding state of the Zn**_1_**Ni**_2_** hierarchical structure, X-ray photoelectron spectroscopy (XPS) analysis was also carried out as shown in Figure 5. For the full range of XPS spectra Figure 5a, the binding energies corresponding to the different energy levels of Zn, Ni, O and C are evidently detected, and the binding energy at 284.6 eV of the C 1s is used as a reference for calibration. Figure 5b displays the XPS spectra of Zn 2p, the measured spectrum shows two strong peaks with binding energy values of 1021.2 eV and 1044.3 eV, respectively, which can be assigned to Zn 2p_3/2_ and Zn 2p_1/2_ peaks [26]. Figure 5c shows the high-resolution spectrum of Ni 2p, including the characteristics peaks and the corresponding satellite peaks. The peaks located at 853.6 eV, 855.6 eV, and 861.1 eV are assigned to Ni2p_3/2_ and its satellite peak. The higher binding energy located at 872.6 eV along with 879.1 eV can be attributed to Ni 2p_1/2_ and its corresponding satellite peak, which indicate the presence of Ni in the product [27,28]. Furthermore, the O 1s spectrum exhibits the two oxygen states of 529.5 eV and 531.2 eV Figure 5d. The lower binding energy peak corresponds to the lattice oxygen of O^2−^ in the oxide (ZnO, NiO), and the higher energy peak is associated with the chemisorbed oxygen species such as O^−^ and O**_2_**^−^ [29].

### 3.2. Gas Sensing Properties of Sensors

**Figure 6:**
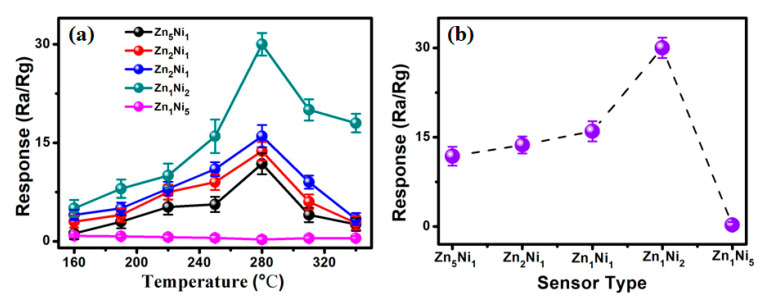
(**a**) Gas response of as-fabricated ZnO/NiO sensor toward 200 ppm NH**_3_** under different working temperatures. (**b**) Relationship of Response vs. Sensor type in presence of 200 ppm NH**_3_** at 280 °C.

It is well-known that the sensor operating temperature can significantly influence its gas sensing properties [30], because it influences the process of oxygen adsorption and desorption on the sensing material. Hence, to optimize the sensor working temperature, gas sensing measurements were conducted on all the as-fabricated 3D Zn_x_Ni_y_-based sensors toward 200 ppm NH**_3_** gas within the working temperature range of 160 to 340 °C, as presented in Figure 6a. Clearly, the gas sensing responses of Zn**_5_**Ni**_1_**, Zn**_2_**Ni**_1_**, Zn**_1_**Ni**_1_** and Zn**_1_**Ni**_2_** sensors increased to their maximum as the working temperature risen to 280 °C, and then decreased with further rise of the working temperature towards 340 °C. Thus, the optimum working temperature could be selected to be at 280 °C. The obtained phenomenon of the gas sensing performance was in good agreement with previous published works [31].

However, for the Zn**_1_**Ni**_5_** based sensor, Figure S1 (Supporting Information S1) clear shows that the sensor response revealed the inverse tendency comparing with other *n*-ZnO/*p*-NiO based sensors within the working temperature range. We attributed the reason to the higher chemical composition of Ni element in Zn**_1_**Ni**_5_** hierarchical nanostructures (as shown in Figure 2e), as it shown typical *p*-type response to NH**_3_** gas [32]. Furthermore, Figure 6b illustrates the response of all the as-developed 3D Zn_x_Ni_y_-based sensors toward 200 ppm NH**_3_** at 280 °C. Obviously, the Zn**_1_**Ni**_2_** sensor demonstrated the highest response of 31.2 toward 200 ppm NH**_3_**, which is much higher than that of Zn**_5_**Ni**_1_** (11.8), Zn**_2_**Ni**_1_** (13.7), Zn**_1_**Ni**_1_** (16) and Zn**_1_**Ni**_5_** (0.26) based sensors. The obtained results unambiguously suggest that the appropriate Zn-to-Ni molar ratio is very advantageous for enhancement gas sensing performances of the 3D *n*-ZnO/*p*-NiO based sensor, and the as-developed Zn**_1_**Ni**_2_** sensor displayed the best gas sensing property towards NH**_3_** sensing.

**Figure 7:**
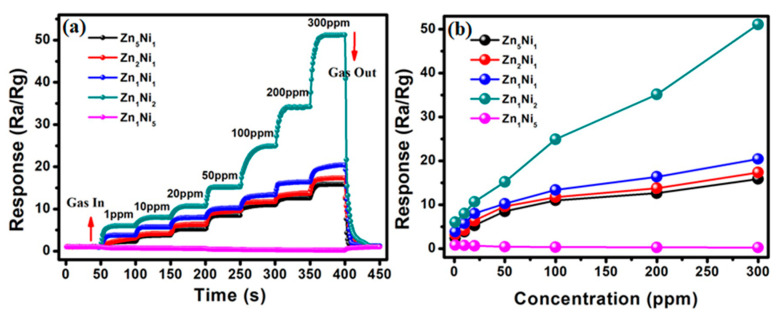
(**a**) Dynamic response of as-developed 3D *n*-ZnO/*p*-NiO based sensors toward high NH**_3_** concentration (1–300 ppm) at 280 °C. (**b**) Gas responses vs. NH_3_ concentrations curves of ZnO/NiO sensors.

The dynamic gas sensing experiments were also conducted on the as-developed 3D Zn_x_Ni_y_-based sensors to investigate their responses to the different NH**_3_** concentrations. Figure 7 depicts the sensor response to various NH**_3_** concentrations from 1 ppm to 300 ppm at the optimum working temperature (280 °C). Evidently, when gas sensors were exposed to 1 ppm of NH**_3_** gas, the sensor responses manifested increasing tendency for Zn**_5_**Ni**_1_**, Zn**_2_**Ni**_1_**, Zn**_1_**Ni**_1_** and Zn**_1_**Ni**_2_**, as shown in Figure 7a. By successively injecting NH**_3_** gas with its concentration increasing from 1 ppm to 300 ppm, the sensor responses rose steeply, and then tended to a stable value, as well as exhibited appropriate step responses. Subsequently, when the 300 ppm of NH**_3_** was removed, their measured responses were quickly recovered to their initial response level, indicating that the as-prepared gas sensors based on 3D *n*-ZnO/*p*-NiO have good response-recovery characteristics. Particularly, the as-fabricated Zn**_1_**Ni**_2_** sensor exhibited the superior dynamic response behavior compared to the other 3D Zn_x_Ni_y_-based sensors. Moreover, the measured sensor responses of Zn**_1_**Ni**_2_** sensor can reach to 6.1 (1 ppm), 8.0 (10 ppm), 10.7 (20 ppm), 15.2 (50 ppm), 24.9 (100 ppm), 34.2 (200 ppm) and 51.2 (300 ppm), respectively. On the other hand, Figure S2 (Supporting Information S2) illustrates the dynamic response of the Zn**_1_**Ni**_5_** based sensor to NH**_3_** at the optimum working temperature (280 °C), and the amplification image revealed that the opposite response could be observed when the sensor was exposed to the different NH**_3_** concentrations. Consequently, the relationship between the gas responses and NH**_3_** concentrations were also presented for as-fabricated Zn_x_Ni_y_ sensors in Figure 7b. Compared with other 3D Zn_x_Ni_y_ hierarchical structures, the obtained results demonstrated that the sensor based on Zn**_1_**Ni**_2_** hierarchical structure presents the maximum response under the same NH**_3_** concentration.

**Figure 8:**
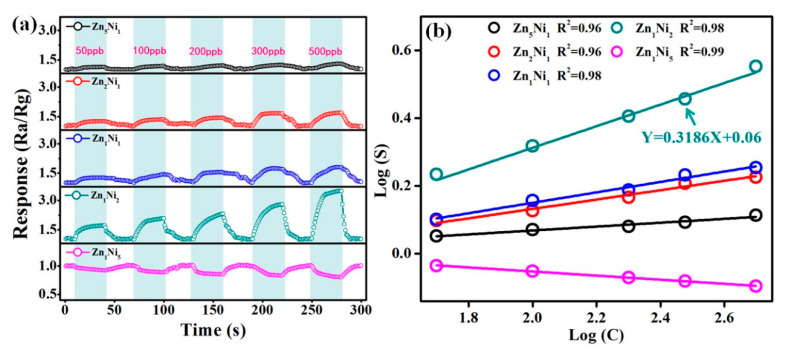
(**a**) Real-time response curves of 3D *n*-ZnO/*p*-NiO sensors toward the low NH**_3_** concentrations from 50 to 500 ppb at 280 °C. (**b**) Corresponding response of linear relationship with logarithmic forms of *n*-ZnO/*p*-NiO sensors.

In order to further evaluate the gas sensing behavior of 3D *n*-ZnO/*p*-NiO based sensors toward the low concentrations of NH**_3_**, the dynamic response-recovery characteristics of sensors were evaluated by their exposure to 50–500 ppb of NH**_3_** gas at the optimum working temperature of 280 °C. Figure 8a illustrates the real-time response curves of the 3D Zn_x_Ni_y_ based sensors toward 50 ppb, 100 ppb, 200 ppb, 300 ppb and 500 ppb target gases, and the sensor responses can recover to the baseline level when fresh air is injected, which suggests good response-recovery and reversibility for the low NH**_3_** concentrations at ppb level. Moreover, the obtained data confirmed that the Zn**_1_**Ni**_2_** sensor still demonstrated better sensing capabilities than the other sensors, revealing its superior NH**_3_** sensing capacity even the ppb level. We speculated that the specific hierarchical nanosheet structure of the *n*-ZnO/*p*-NiO could produce much more contact sites on its surface. Thus, many NH**_3_** molecules will be adsorbed on the surface of the heterostructured sensing materials and oxidized by oxygen species at contact sites even at very low NH**_3_** concentration. Additionally, Figure 8b depicts the corresponding response of the linear relationships in logarithmic forms of the 3D Zn_x_Ni_y_ sensors (and the relationships of NH**_3_** concentration vs. response is shown in Figure S3 (Supporting Information S3)). The obtained relationship for the Zn**_1_**Ni**_2_** sensor can be defined as the linear equation: *Y*  =  0.318 *X*  +  0.06 (R^2^ =  0.98), and the slope of the straight line revealed that the sensor has high sensitivity even in the low concentrations of NH**_3_**. Moreover, according to the measured experimental results, the linear correlation coefficients (R^2^) are close to 1 for all the as-prepared 3D *n*-ZnO/*p*-NiO sensors, which indicated good linear relationships towards NH**_3_**.

**Figure 9:**
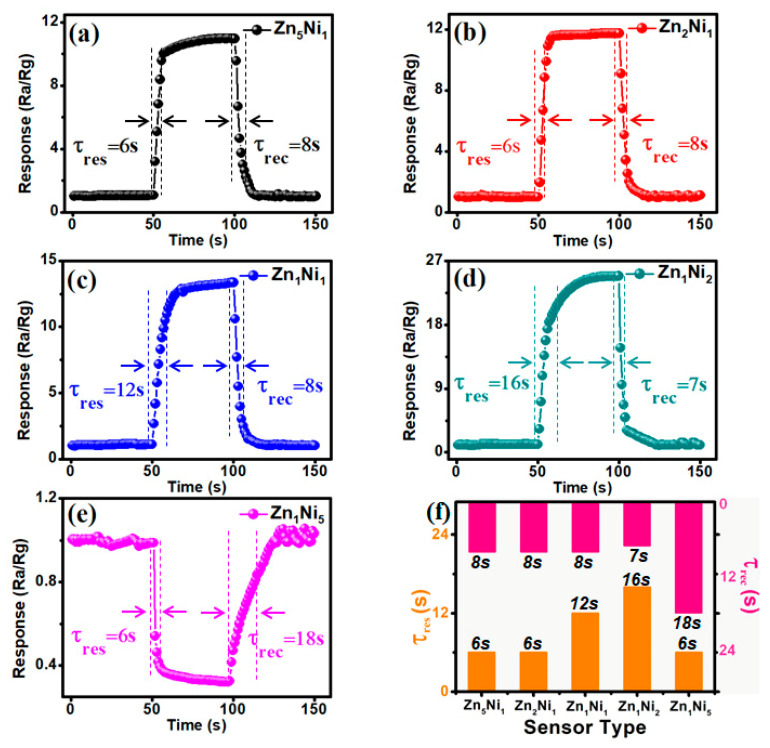
Dynamic sensing transient of *n*-ZnO/*p*-NiO based sensors toward 100 ppm NH**_3_**: Zn**_5_**Ni**_1_** (**a**), Zn**_2_**Ni**_1_** (**b**), Zn**_1_**Ni**_1_** (**c**), Zn**_1_**Ni**_2_** (**d**) and Zn**_1_**Ni**_2_** (**e**). (**f**) Response and recovery times of *n*-ZnO/*p*-NiO sensors.

The response/recovery characteristic is one of the most important features for the NH**_3_** gas sensor for practical applications. Figure 9a–e represent the dynamic response and recovery curves of as-prepared Zn_x_Ni_y_-based sensors toward 100 ppm NH**_3_** at the optimum working temperature of 280 °C. As summarized in Figure 9f, the measured response/recovery times (τ_res_/τ_rec_) of Zn**_5_**Ni**_1_**, Zn**_2_**Ni**_1_**, Zn**_1_**Ni**_1_**, Zn**_1_**Ni**_2_** and Zn**_1_**Ni**_2_** sensors were 6/8 s, 6/8 s, 12/8 s, 16/7 s and 6/18 s, respectively. Although the recovery time for Zn**_1_**Ni**_2_** sensor was calculated to be about 16 s, the measured recovery time is only 7 s towards 100 ppm NH**_3_**, which is a quite short recovery time among the as-developed *n*-ZnO/*p*-NiO sensors. Therefore, the obtained results demonstrated that the Zn**_1_**Ni**_2_** sensor is indeed possesses good response-recovery characteristic for NH**_3_** detection. In addition, the sensing performance of the as-developed Zn**_1_**Ni**_2_**-based and most of the previously reported ZnO or NiO based NH**_3_** gas sensors are summarized in Table 1. When exposed to 100 ppm gas environment, the Zn**_1_**Ni**_2_** sensor demonstrated outstanding sensitivity and response/recovery characteristics, as well as very low *ppb* detection limit.

**Figure 10:**
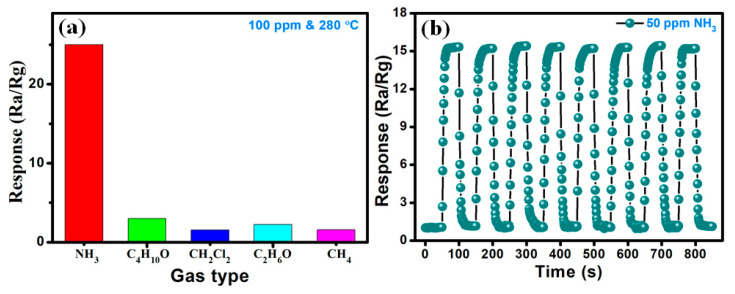
(**a**) Selectivity of Zn**_1_**Ni**_2_** gas sensor toward various gases (100 ppm) at 280 °C. (**b**) Reversibility of Zn**_1_**Ni**_2_** for 50 ppm NH**_3_**.

Figure 10a illustrates the selectivity test results for the Zn**_1_**Ni**_2_** based NH**_3_** sensor, when it was exposed to 100 ppm of NH**_3_** and other potential interfering gases including 1-butanol (C**_4_**H**_10_**O), dichloromethane (CH**_2_**Cl**_2_**), ethanol (C**_2_**H**_6_**O), and methane (CH**_4_**) at the optimal working temperature of 280 °C. The experimental results display that the Zn**_1_**Ni**_2_** gas sensor exhibited the highest response (25) towards NH**_3_** compared with C**_4_**H**_10_**O (3.01), CH**_2_**Cl**_2_** (1.55), C**_2_**H**_6_**O (2.22) and CH**_4_** (1.58), which demonstrates an excellent selectivity of the Zn**_1_**Ni**_2_** sensor. In addition to selectivity, the repeatability is another important parameter for any gas sensor. Thus, Figure 10b shows another test result, when the sensors was consistently exposed to the cycling changes of NH**_3_** concentrations. It displays the response-recovery curve of the Zn**_1_**Ni**_2_** sensor at the presence/absence of 50 ppm NH**_3_** for eight cycles at 280 °C. We can clearly observe that the sensor response maintains around 15 after the reversible cycles, illustrating good reversibility and reproducibility of the sensor performance.

**Figure 11:**
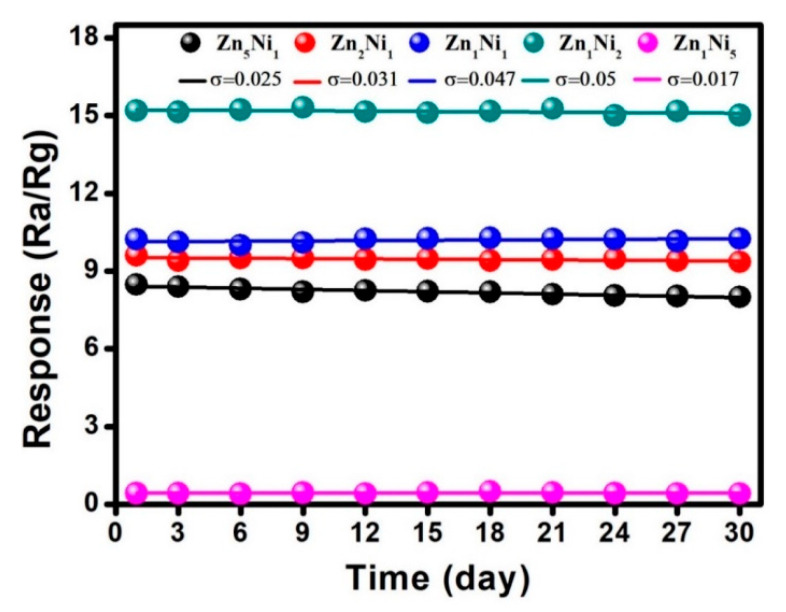
Long-term stability of as-prepared 3D *n*-ZnO/*p*-NiO based sensors toward 50 ppm NH**_3_** at 280 °C.

Apart from investigation of selectivity and reproducibility characteristics, long-term stability of as-prepared *n*-ZnO/*p*-NiO based sensors was also investigated at the laboratory conditions. Figure 11 plots the collected response values when the sensors were exposed to 50 ppm NH**_3_** for 30 consecutive days. Obviously, there was no significant fluctuation in the response curves for the *n*-ZnO/*p*-NiO based sensors, and specifically, the response for Zn**_1_**Ni**_2_** sensor showed only 5.0% fluctuation over 30 days, suggesting the good long-term stability for NH**_3_** detection.

### 3.3. Gas Sensing Mechanism

**Figure 12:**
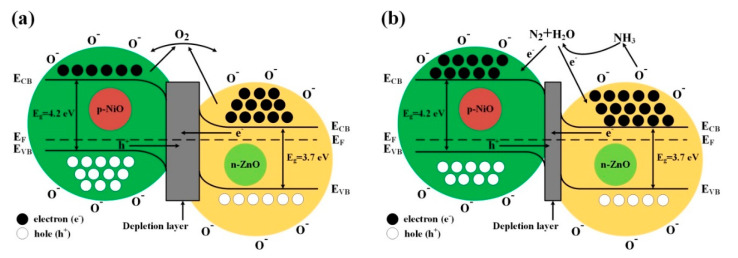
Proposed band structure model for 3D *n*-ZnO/*p*-NiO heterojunction (**a**) in air and (**b**) in NH**_3_**. (E_CB_, lower level of conduction band; E_F_, Fermi level; E_VB_, upper level of valence band).

For most of MOS-based gas sensors, the gas sensing mechanism can be explained by the resistance change on the surface of the sensing electrode material which is caused by adsorption and desorption of the target gas molecules [43]. The detailed sensing mechanism and the surface reaction of the 3D *n*-ZnO/*p*-NiO based sensor were depicted in Figure 12. When the as-prepared 3D *n*-ZnO/*p*-NiO sensor is exposed to air Figure 12a, the highly electronegative of oxygen molecules could be easily absorbed on the surface of sensing material by trapping free electrons, and then form different chemisorbed oxygen species (O^−^ in this experiment) under the various working temperatures (including O**_2_**^−^ (T < 100 °C), O^−^ (100 < T < 300 °C), and O^2−^ (T > 300 °C) [44,45]. The loss of electrons will lead to a depletion layer and significantly increased resistance on the surface of *n*-type ZnO. In contrast, for the *p*-type NiO, the process will form a hole-accumulation layer and decrease the resistance. Their synergistic effect results in the change of sensor resistance. For instance, when NH**_3_** (reducing gas) is injected, O^−^ will react with NH**_3_** molecules and release electrons into the sensing electrode material, eventually leading to a resistance variation and sensing response Figure 12b. The gas sensing reaction for the *n*-ZnO/*p*-NiO hierarchical structures towards NH**_3_** detection can be described by the following equations:

*O***_2_**_(gas)_—>2*O*_(adsorbed)_ (1)

*O* + *e*^─^—> *O*^─^ (2)

2*NH***_3_** + 3 *O*^─^—> *N***_2_** + 3*H***_2_***O*+3 *e*^─^ (3)

Meanwhile, the excellent gas sensing properties of the *n*-ZnO/*p*-NiO gas sensor could be attributed to two aspects: on one hand, the morphological features exhibited the specific hierarchical nanosheets structure, which facilitated the adsorption of NH**_3_** molecules on the surface of hetero-structured sensing electrode and produces much more contact sites. On the other hand, the formation of *p*-*n* heterojunctions on the interface between the *n*-type ZnO and *p*-type NiO can contribute significantly to the improvement of the sensor responses. When the ZnO intimately contacts with the NiO, an internal self-built electrical field at the interface will be formed via charge carries diffusion due to their different work functions, electron affinities and band gaps [46]. In the electrical field, the electrons transfer from ZnO to NiO, and holes move in the opposite direction until the systems achieve equalization at the Fermi level, as displayed in Figure S4 (Supporting Information S4) [24,47]. The process will lead to development of the potential barrier at the heterojunctions as the band bending and a wider depletion layer appears at their interface. For the Zn**_1_**Ni**_2_** sample, it displays n-type semiconductor gas senor which makes the electrons as majority carriers. These free electrons will be trapped by oxygen in air and lead to the increase of depletion layer width at *p*-*n* junction Figure 12a, thus it will further increase the resistance of the Zn**_1_**Ni**_2_** than the pure ZnO. Correspondingly, the measured resistance of Zn**_1_**Ni**_2_** will higher than pure ZnO. When the Zn**_1_**Ni**_2_** sensor is exposed to NH**_3_** gas, the ammonia molecules will be oxidized by oxygen species and the trapped electrons will be released again. During the process, the depletion layer width at *p*-*n* junction is decreased and so that the resistance value drops drastically. Therefore, the depletion layer which formed at *p*-*n* junction is the main contributor to the best sensing performance of Zn**_1_**Ni**_2_** sensor. Noteworthy, with the amount of NiO was increased, the gas sensor shown changes from *n*- to *p*-type transition. For example, for the *n*-type sensors (Zn**_5_**Ni**_1_**, Zn**_2_**Ni**_1_**, Zn**_1_**Ni**_1_** and Zn**_1_**Ni**_2_**), the resistance will increase in air and decrease in NH**_3_**, whereas for the p-type sensor (Zn**_1_**Ni**_5_**) the behavior is quite an opposite.

## 4. Conclusions

In summary, a series of 3D flower-like *n*-ZnO/*p*-NiO heterojunction composites with the different Zn-to-Ni ratios were synthesized (Zn_x_Ni_y_, x:y = 5:1, 2:1, 1:1, 1:2, 1:5) towards the development of sensing electrode with superior NH**_3_** sensing performance by a facile hydrothermal method. Their structural and morphological properties were comprehensively investigated by XRD, SEM, TEM and XPS techniques, confirming that the 3D flower-like *n*-ZnO/*p*-NiO structures were successfully created. The sensing performances of the Zn_x_Ni_y_ heterostructures towards NH**_3_** sensing were analyzed under various working temperatures (160–340 °C) and showed different gas-sensing properties. Specifically, the fabricated NH**_3_** sensor based on Zn**_1_**Ni**_2_** displayed the best sensing capabilities towards NH**_3_** detection at the established optimum working temperature of 280 °C. Moreover, this sensor exhibited the high response of 25 and fast response/recovery time of 16 s/7 s toward 100 ppm NH**_3_**, as well as the low detection limit of 50 ppb, good selectivity and long-term stability. Therefore, the present study has successfully demonstrated the vital role of the *p*-*n* heterojunctions for the enhancement of gas sensing properties, confirmed that the 3D *n*-ZnO/*p*-NiO nanocomposites are sensing material candidate for constructing NH**_3_** sensors with promising application.

## Figures and Tables

**Table 1:** Comparison of the sensing performance of various gas sensors toward NH**_3_**.

Sensing Materials	C (ppm)	T (°C)	Response	*τ_res_*/*τ_rec_* (s)	LOD (ppm)	Ref.
NiO	150	300	141.3%	-/-	25	[33]
NiO-MoS_2_	20	RT	79	160/117	0.25	[6]
Pt/NiO	1000	300	1278	15/76	0.01	[34]
ZnO nanorod	100	650	22.6	-/-	20	[35]
ZnO thin films	600	150	57.5	660/160	50	[36]
Co-ZnO	100	RT	3.48	-/-	15	[37]
3%Er-doped ZnO	120	RT	97%	120/10	--	[38]
ZnO-CNTs	50	RT	171(*I_gas_/I_air_*)	18/35	0.2	[39]
CNTs/rGO/ZnO	10	RT	~2.25	55/116	5	[40]
WO_3_/ZnO	300	250	25	60/50	25	[41]
ZnO/NiO nanofibers	100	RT	6	-/-	50	[42]
Zn_1_Ni_2_	100	280	25	16/7	0.05	This work

C: Gas concentration; T: Operating temperature; *τ_res_*/*τ_rec_*: Response/recovery time; LOD: Limit of detection; RT: Romm Temperature.

## Supplementary Materials

The following are available online at https://www.mdpi.com/1424-8220/20/17/4754/s1, Figure S1: Gas response of the Zn**_1_**Ni**_5_** toward 200 ppm NH**_3_** under the different working temperatures, Figure S2: Dynamic response of the Zn**_1_**Ni**_5_** to 1–300 ppm concentrations of NH**_3_** at 280 °C, Figure S3: Response of the 3D *n*-ZnO/*p*-NiO with the different Zn-to-Ni molar ratio to 50–500 ppb NH**_3_** concentrations at 280 °C, Figure S4: Energy band structure of p-type NiO and n-type ZnO before and after contact.
