# Supplementary material for: NH3 Sensor Based on 3D Hierarchical Flower-Shaped n-ZnO/p-NiO Heterostructures Yields Outstanding Sensing Capabilities at ppb Level
Source: Sensors (Basel). 2020 Aug 22;20(17):4754. doi: 10.3390/s20174754 (PMC7506851; doi:10.3390/s20174754)
Supplement: Supplementary file 1 [file sensors-20-04754-s001.pdf]

Supporting information

NH_3 sensor based on 3D hierarchical flower-shaped ***n*-ZnO/*p*-NiO** heterostructures yields outstanding sensing capabilities at ppb level

*Zhenting Zhao[a, b], Haoyue Yang[b], Zihan Wei[c], Yan Xue[b], Yongjiao Sun[b], Wenlei Zhang[b], Pengwei Li[b], Weiping Gong[a], Serge Zhuiykov[c] and Jie Hu[b *]*

[a] *Guangdong Provincial Key Laboratory of Electronic Functional Materials and Devices, Huizhou University, Huizhou 516001, Guangdong, China*

[b] *Center of Nano Energy and Devices, College of Information and computer, Taiyuan University of Technology, Taiyuan 030024, Shanxi, China*

[c] *Ghent University Global Campus, Department of Solid State Science, Faculty of Science, 119 Songdomunhwa-ro, Yeonsu-gu, Incheon 21985, South Korea*

**Correspondence: hujie@tyut.edu.cn*

S1: Gas response of the Zn_1Ni_5 under different working temperatures

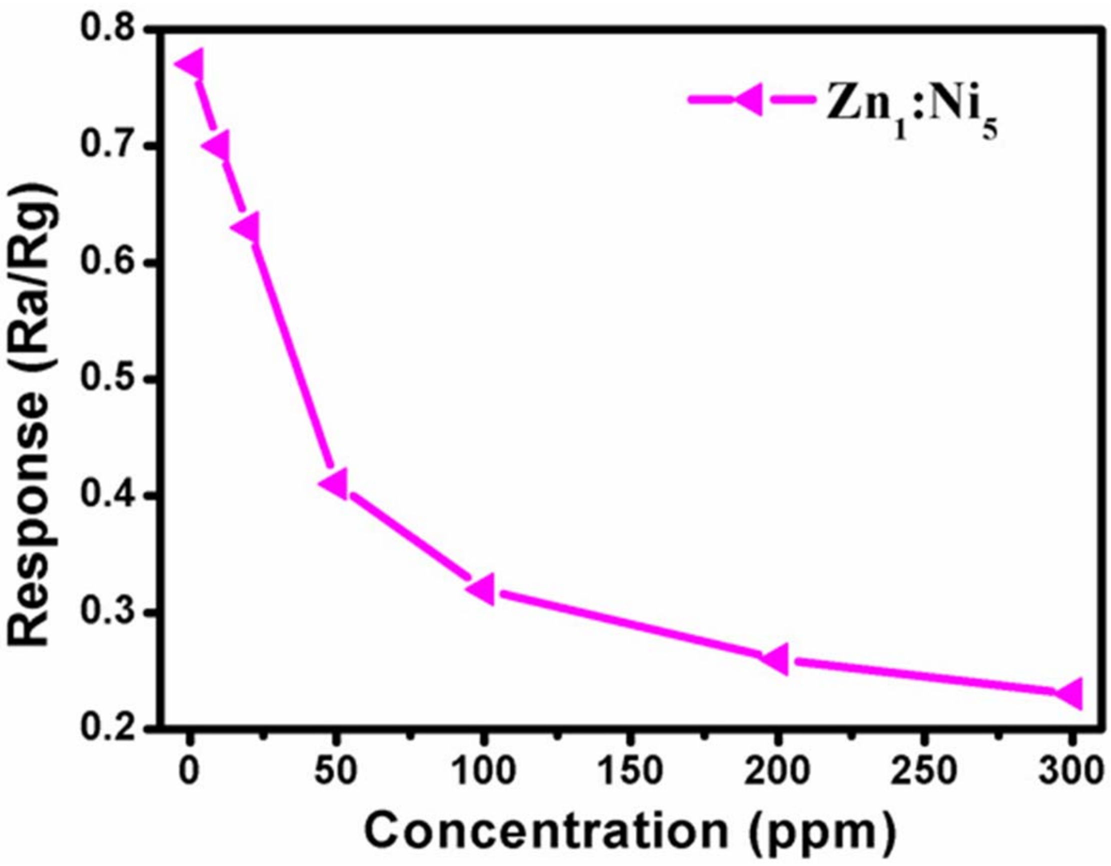

Figure. S1 Gas response of the Zn_1Ni_5 toward 200 ppm NH_3 under the different working temperatures.

S2: Dynamic response of the Zn_1Ni_5 in presence of 1-300 ppm concentrations of NH_3

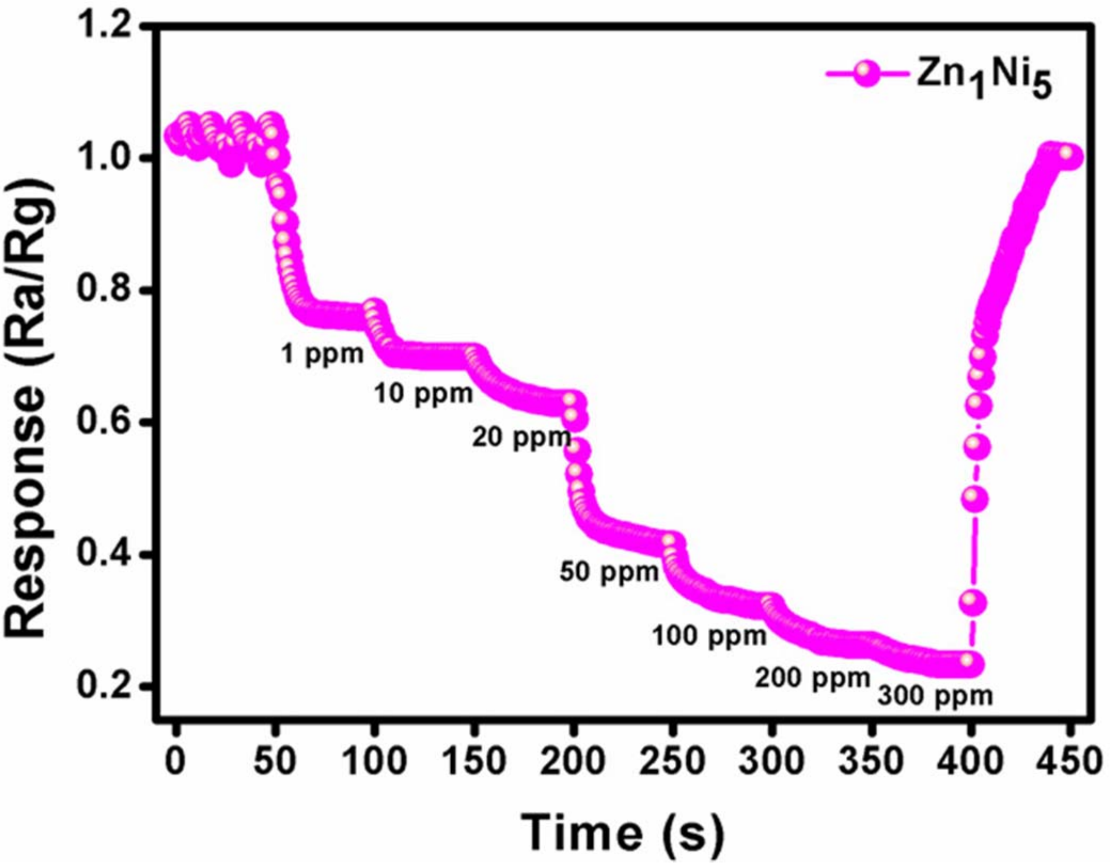

Figure. S2 Dynamic response of the Zn_1Ni_5 to 1-300 ppm concentrations of NH_3 at 280 °C.

S3: Relationship of NH_3 concentration vs. response

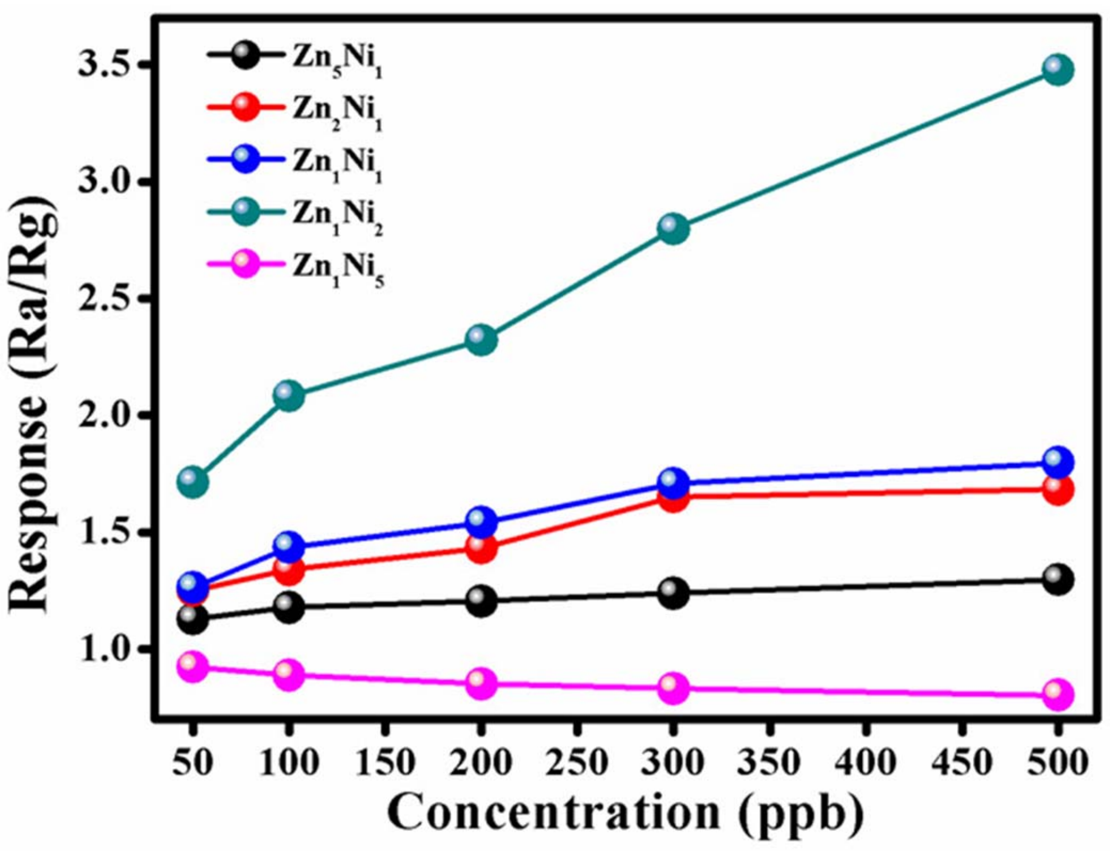

Figure. S3 Response of the 3D *n*-ZnO/*p*-NiO with the different Zn-to-Ni molar ratio to 50-500 ppb NH_3 concentrations at 280 °C.

S4: Energy band structure of p-type NiO and n-type ZnO before and after contact

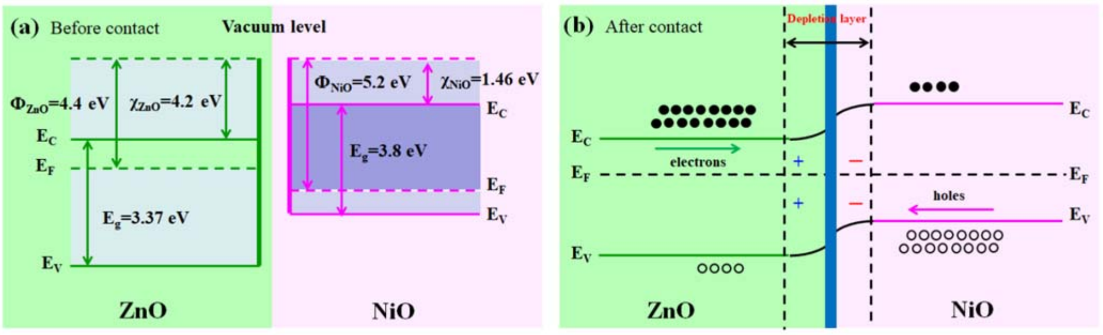

Figure. S4 Schematic diagram of the energy band structure of p-type NiO and n-type ZnO before (a) and after (b) contact.
